# Biotransformation of Pristine and Oxidized Carbon Nanotubes by the White Rot Fungus *Phanerochaete chrysosporium*

**DOI:** 10.3390/nano9091340

**Published:** 2019-09-19

**Authors:** Qiang Ma, Ailimire Yilihamu, Zhu Ming, Shengnan Yang, Mengyao Shi, Bowei Ouyang, Qiangqiang Zhang, Xin Guan, Sheng-Tao Yang

**Affiliations:** College of Chemistry and Environment Protection Engineering, Southwest Minzu University, Chengdu 610041, China; qiang9322@outlook.com (Q.M.); almira1250@outlook.com (A.Y.); 15982889615@163.com (Z.M.); 18641460881@163.com (S.Y.); 18328095288@163.com (M.S.); ouyang13299547661@163.com (B.O.); zqq950526@163.com (Q.Z.); GX491039743@163.com (X.G.)

**Keywords:** carbon nanotubes, white rot fungi, decomposition, oxidative degradation, nano-biosafety

## Abstract

Carbon nanomaterials are widely studied and applied nowadays, with annual production increasing. After entering the environment, the complete degradation of these carbon nanomaterials by microorganisms is proposed as an effective approach for detoxification and remediation. In this study, we evaluated the degradation of pristine multiwalled carbon nanotubes (p-MWCNTs) and oxidized multiwalled carbon nanotubes (o-MWCNTs) by the white rot fungus *Phanerochaete chrysosporium*, which is a powerful decomposer in the carbon cycle and environmental remediation. Both p-MWCNTs and o-MWCNTs were partially oxidized by *P. chrysosporium* as indicated by the addition of oxygen atoms to the carbon skeleton in the forms of C=O and O–H bonds. The fungal oxidation led to the shortening of MWCNTs, where precipitated o-MWCNTs showed more short tubes. During the transformation, the defects on the tubes became detached from the carbon skeleton, resulting in decreases of the *I*_D_/*I*_G_ (intensity of D-band/ intensity of G-band) values in Raman spectra. The transformation mechanism was attributed to the enzymatic degradation by laccase and manganese peroxidase excreted by *P. chrysosporium*. The results collectively indicated that MWCNTs could be transformed by *P. chrysosporium*, but complete degradation could not be achieved in a short time period. The implications on the environmental risks of carbon nanomaterials are discussed.

## 1. Introduction

Carbon nanomaterials have been the most attractive nanomaterials since the discovery of fullerene, carbon nanotubes (CNTs), and graphene [1,2,3]. Carbon nanomaterials find various important applications in many areas, such as electronics, environmental analysis and remediation, biomedicine, energy, healthcare, and phonics [1,2,3]. The annual production of carbon nanomaterials is growing rapidly, arousing environmental risk concerns [4,5]. Many studies have highlighted the environmental hazards of carbon nanomaterials. For example, we found that graphene materials induced damage to plant roots and inhibited the photosynthesis of leaves [6,7]. Cimbaluk et al. reported that CNTs induced neurotoxicity to fish through oxidative stress [8]. Therefore, the effective remediation of carbon nanomaterials in the environment is becoming a crucial issue for the healthy development of carbon nanomaterials [9,10].

Microorganisms are the most important decomposers in the biogeochemical cycles [11]. The powerful decomposition activity of microorganisms has already been adopted in the remediation of traditional pollutants, such as oil, pesticides, urban wastewater, and industrial effluents [12]. Inspired by this, many researchers have proposed that carbon nanomaterials could be degraded by microorganisms to eliminate the potential hazards [13,14,15]. The Fenton reaction that generates hydroxyl radicals was the first considered demonstration, which was performed in the Fe^2+^–H_2_O_2_ system and then the horseradish peroxidase (HPR)–H_2_O_2_ system [16,17]. Biodegradation of CNTs was reported by Allen et al. using the HPR–H_2_O_2_ system after 8 weeks [16]. Similar observations were also reported in graphene [18], graphene oxide (GO) [19], and other carbon nanomaterials [15]. Only 0.5% of ^14^C-CNTs and ^13^C-CNTs were mineralized after a 30 day (d) treatment in the HRP–H_2_O_2_ system [20]. Despite the contradictory results of solution degradation, highly active microorganisms have been selected to degrade carbon nanomaterials [21,22]. However, contradictory results have also been obtained by different groups. For example, Navarro et al. showed that less than 0.025% of ^14^C_60_ and 3% of ^14^C_60_-OH were completely mineralized as CO_2_ in soil after 55 d [23]. Similarly, ^14^C_60_ was reported to be resistant to mineralization in a plant culture system for 2 years [24]. On the other hand, Berry et al. found that up to 56.26% of ^13^C_60_-OH was mineralized in contrasting agricultural soils [25]. Elgrabli et al. reported the fast degradation of CNTs in macrophages [26], while Sato et al. observed stable CNTs in microphages of the liver after 2 years 3 months [27].

Among these microorganisms, white rot fungi have been attracting more attention recently [28]. White rot fungi produce ligninolytic enzymes, namely laccase (Lac), manganese peroxidase (MnP), and ligninase, and then the enzymes catalyze the generation of oxidative radicals to attack lignin or pollutants. The advantages of white rot fungi include the production of highly reactive radicals, nonselectivity for substrates, and indirect contact between the carbon nanomaterials and the enzymes, protecting the activity of the enzymes. Protein level studies indicated that ligninolytic enzymes could degrade carbon nanomaterials [29,30,31]. However, for in vivo degradation by white rot fungi, there is very limited information available. Only two studies concerned the mineralization rate. White rot fungi (*Phlebia tremellosa* and *Trametes versicolor*) could very slowly metabolize and degrade ^13^C_60_-OH into CO_2_ (less than 3%) and fatty acids (less than 0.1%) after 32 weeks. For more stable CNTs, the mineralization rate was less than 0.1% after 168 d of degradation by *T. versicolor* [32]. The degradation of carbon nanomaterials in white rot fungi should be further investigated, because oxidation and partial degradation may occur prior to complete mineralization.

In this study, we compared the degradation of pristine multiwalled carbon nanotubes (p-MWCNTs) and oxidized multiwalled carbon nanotubes (o-MWCNTs) by the white rot fungus *Phanerochaete chrysosporium*. The morphologies of MWCNT samples were checked under transmission electron microscopy (TEM) and scanning electron microscopy (SEM) after transformation. The chemical changes were evaluated by infrared (IR) spectroscopy and X-ray photoelectron spectroscopy (XPS). The defect contents were investigated by Raman spectroscopy. The enzymatic activities of *Phanerochaete chrysosporium* were assayed to reflect the transformation mechanism. The results were compared with the literature results to analyze the potential of degradation of carbon nanomaterials by white rot fungi. The implications on the remediation of carbon nanomaterials by microorganisms are discussed.

## 2. Materials and Methods

### 2.1. Materials

P-MWCNTs were bought from Shenzhen Nanotech Port, Ltd., Shenzhen, China (purity of >97 wt %, length >5 µm, diameter of 10–20 nm). P-MWCNTs were oxidized by H_2_SO_4_/HNO_3_ as described in our previous report [33]. Both p-MWCNTs and o-MWCNTs were characterized by TEM (Tecnai G2 20, FEI, Hillsboro, OR, USA), XPS (ESCALAB 250XI, Kratos, Manchester, UK), IR (Magna-IR 750, Nicolet, Alexandria, LA, USA), and Raman spectroscopy (inVia, Renishaw, Wotton-under-Edge, UK) before use.

### 2.2. Degradation of MWCNTs

Fungal strain ACCC 30942 (*P. chrysosporium*) was bought from the Agricultural Culture Collection of China. The culture medium recipe and culture protocols were the same as described in our previous report [32]. The culture medium was supplemented with p-MWCNTs or o-MWCNTs at the concentrations of 1.0 mg/mL for *P. chrysosporium* culture. The pH values of the culture media were adjusted to 4.5 with NaOH or HCl aqueous solutions (PB10, Sartorius Co., Germany). To each flask was added 100 mL medium, which was inoculated with 5.0 × 10^7^ spores. The flasks were incubated on a thermostat shaker (CHA-S, Jintan Hankang Electronic Co., Jintan, China) at 150 rpm and 37 °C for 3 d, 7 d, 14 d, and 30 d before measurements were taken.

To separate the CNT samples, the fungus balls were collected by tweezers at designed time points (0–30 d). The CNTs could be divided into two parts: CNTs wrapped in the fungal balls (CNTs wrapped) and CNTs precipitated in the medium (CNTs precipitated). The samples were sonicated with an ultrasonic cell disruptor for 5 min (30 s sonication with 8 s interval each time). To lyse the attached fungal mycelia, the CNT samples were incubated in 100 g/L (sodium dodecyl sulfate and 10 mM CaCl_2_ at 60 °C for 12 h. After the incubation, the CNT samples were filtered and put into 2.7 mM HCl at 60 °C for another 24 h to lyse the proteins. The residues were washed first with ethanol and then with water, each for three times. The CNT samples were lyophilized for characterization by multiple techniques, including XPS, IR, TEM, and Raman spectroscopy.

For ultrastructural observation, the fresh fungus balls were fixed by 2.5% glutaraldehyde overnight and post-fixed in 1% osmium tetroxide. The samples were further dehydrated by a graded alcohol series and embedded in epoxy resin. Thin sections were prepared by an ultramicrotome and post-stained with uranyl acetate and lead citrate for TEM observations.

### 2.3. Enzyme Activities

For enzyme activity measurements, *P. chrysosporium* was exposed to p-MWCNTs and o-MWCNTs at 1 mg/mL for 3 d, 7 d, 14 d, and 30 d. At designed time points, the fungus balls were filtered out for the filtrate collection by filter paper. The Lac activity was measured using 2,2′-azino-bis(3-ethylbenz-thiazoline-6-sulfonate) (ABTS) as the substrate. Briefly, the filtrate was diluted with the buffer to allow the proper kinetics based on the pre-evaluations. Then, 1.0 mL of diluted filtrate, 0.2 mL of 0.5 mM ABTS, and 2.7 mL of 0.1 M sodium acetate buffer (pH 4.8) were mixed and the absorbance was immediately monitored at 420 nm (UV1600, Shanghai Mapada Instruments Co., Shanghai, China). The initial slope was used for the Lac activity calculation.

For MnP activity assay, the same filtrate was diluted to allow the moderate kinetics according to premeasurements. Then, to 0.4 mL of diluted filtrate was added 0.1 mL of 1.6 mM MnSO_4_ and 3.4 mL of 50 mM sodium lactate buffer (pH 4.5). The reaction was triggered by 0.1 mL of 1.6 mM H_2_O_2_. The absorbance at 240 nm was monitored to obtain the initial slope for MnP activity calculation.

### 2.4. Statistical Analysis

The quantitative data were expressed as the average mean of individual observations with standard deviation (mean ± SD). Significance was calculated by the Student’s *t*-test method. The difference was considered as statistically significant at *p* < 0.05.

## 3. Results and Discussion

### 3.1. TEM Observation of MWCNTs after Transformation

**Figure 1:**
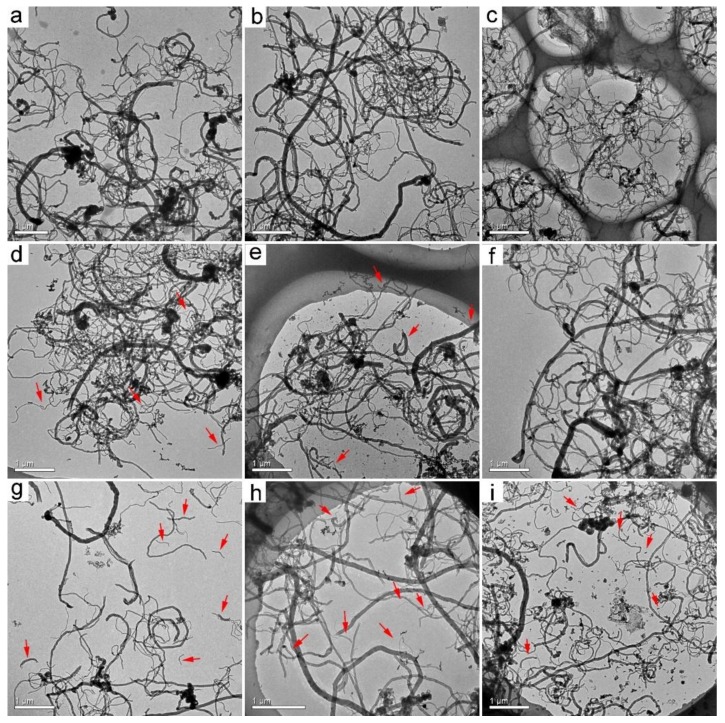
TEM images of pristine multiwalled carbon nanotubes (p-MWCNTs) after the incubation with *P. chrysosporium*. (**a**) As-prepared p-MWCNTs; (**b**) wrapped p-MWCNTs at 3 d; (**c**) wrapped p-MWCNTs at 7 d; (**d**) wrapped p-MWCNTs at 14 d; (**e**) wrapped p-MWCNTs at 30 d; (**f**) precipitated p-MWCNTs at 3 d; (**g**) precipitated p-MWCNTs at 7 d; (**h**) precipitated p-MWCNTs at 14 d; (**i**) precipitated p-MWCNTs at 30 d. Shortened p-MWCNTs are indicated by red arrows.

**Figure 2:**
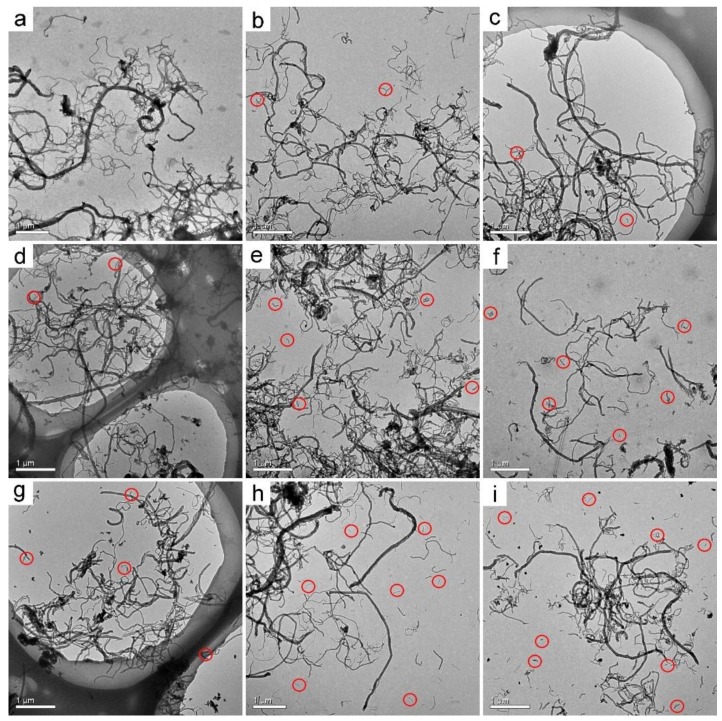
TEM images of oxidized MWCNTs (o-MWCNTs) after the incubation with *P. chrysosporium*. (**a**) As-prepared o-MWCNTs; (**b**) wrapped o-MWCNTs at 3 d; (**c**) wrapped o-MWCNTs at 7 d; (**d**) wrapped o-MWCNTs at 14 d; (**e**) wrapped o-MWCNTs at 30 d; (**f**) precipitated o-MWCNTs at 3 d; (**g**) precipitated o-MWCNTs at 7 d; (**h**) precipitated o-MWCNTs at 14 d; (**i**) precipitated o-MWCNTs at 30 d. Shortened o-MWCNTs are circled in red.

In our previous report, we demonstrated that both p-MWCNTs and o-MWCNTs had low toxicity to *P. chrysosporium* at 1.0 mg/mL [33]. In this study, both MWCNT samples did not inhibit the fungus’ growth. Thus, the high concentration of 1.0 mg/mL was adopted that allowed the separation of more MWCNTs for characterization. At low CNT concentrations, the separation was difficult and less efficient. The separation protocol was the same as in our previous study, which did not change the properties of the carbon nanomaterials. Before transformation by *P. chrysosporium*, the tubular structure of p-MWCNTs was presented under TEM (Figure 1a). The lengths of p-MWCNTs were all longer than 5 µm. There was no apparent change in wrapped p-MWCNTs at 3 d and 7 d (Figure 1b,c). At 14 d, there was a very small number of shorter MWCNTs (<1 µm) observed under TEM (Figure 1d). The shorter ones (<1 µm) became more numerous at 30 d (Figure 1e). The shortening of p-MWCNTs was more obvious for the precipitated ones. Short precipitated p-MWCNTs were easily recognized at 7 d and 14 d (Figure 1g,h). The main population of the precipitated p-MWCNTs was shorter at 30 d (Figure 1i). A similar phenomenon was observed during the transformation of o-MWCNTs. The as-prepared o-MWCNTs had long fibers and short ones (about 3 µm). Even at 3 d, there were shortened o-MWCNTs in both wrapped o-MWCNTs (Figure 2b) and precipitated o-MWCNTs (Figure 2f), as indicated by the red circles (<500 nm). Over time, the shortened o-MWCNTs became more numerous and the lengths were less than 200 nm. Again, the precipitated o-MWCNTs were transformed more, with more short tubes (<200 nm) found under TEM. The shortening of MWCNTs should be regarded as the cutting of tubes by *P. chrysosporium* during the transformation. It should be noted that the destruction of the tubular structure was not observed in the four groups.

The shortening of CNTs during biodegradation is a widely reported phenomenon in the literature. Enzymatic reaction was shown to be efficient in cutting CNTs. Allen et al. used the HRP–H_2_O_2_ system to degrade single-walled CNTs (SWCNTs). After 12 weeks, the SWCNTs were largely shortened and some of them lost the typical fibrous structure of SWCNTs [16]. Flores-Cervantes et al. reported the shortening of CNTs in HRP–H_2_O_2_ degradation evaluations, but no complete degradation was observed according to TEM, SEM, and isotope labeling [20]. Russier et al. observed the shortening and complete destruction of o-MWCNTs and o-SWCNTs under TEM after degradation in the HRP–H_2_O_2_ system [34]. Bhattacharya et al. observed the shortening of oxidized SWCNTs after the degradation by lactoperoxidase [35]. Zhang et al. evidenced the degradation of pristine SWCNTs by MnP under TEM, while Lac did not change the fibrous structure of SWCNTs [36]. Microorganisms and mammalian cells are also capable of cutting CNTs. You et al. observed shorter and thinner nanotubes and highly disordered tubular structure with kinks and bends after the incubation of MWCNTs with *Mycobacterium vanbaalenii* PYR-1 [37]. Kagan et al. counted the lengths of SWCNTs after the incubation with macrophages under TEM. The SWCNT length distribution shifted toward shorter lengths after 72 h of incubation, and the fibrous structure of SWCNTs was retained [38]. Nunes et al. observed shortened MWCNTs in brain cortex by TEM [39]. Overall, the shortening of MWCNTs by *P. chrysosporium* was reasonable, which was related to the oxidation of carbon atoms in the tubular skeleton.

### 3.2. XPS Analyses of MWCNTs after Transformation

**Figure 3:**
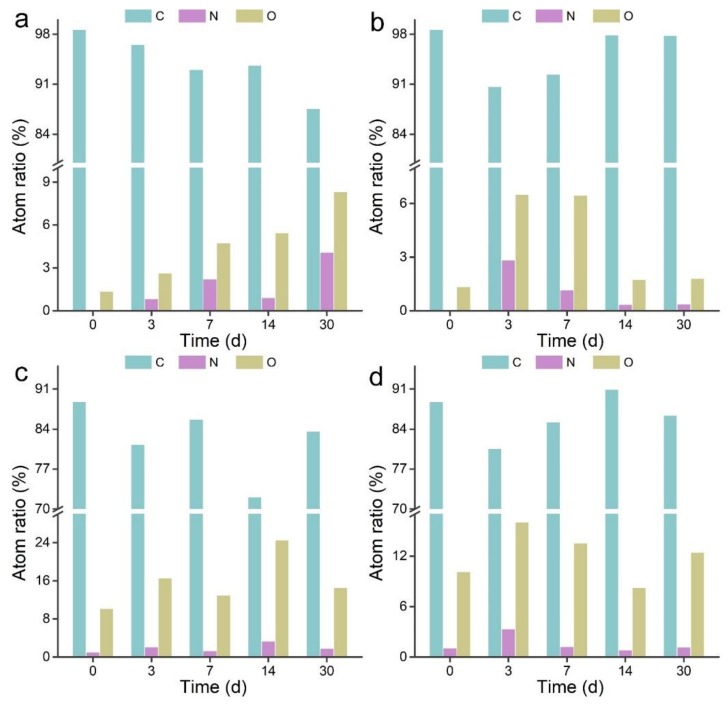
Chemical components of MWCNTs after the incubation with *P. chrysosporium*. (**a**) Wrapped p-MWCNTs; (**b**) precipitated p-MWCNTs; (**c**) wrapped o-MWCNTs; (**d**) precipitated o-MWCNTs.

XPS was adopted to analyze the elemental components and chemical states of MWCNTs before and after the transformation. As shown in Figure 3a, the C content of wrapped p-MWCNTs decreased along with the increase of O content during the transformation. The N content showed an increasing trend, except for the 14 d sample. This suggested that wrapped p-MWCNTs were gradually oxidized by *P. chrysosporium*. For the precipitated p-MWCNTs, the C content decreased at 3 d and increased thereafter. The O and N contents showed maximum levels at 3 d and decreased at 7 d to 30 d. A possible reason for this was that the outside layer was oxidized at 3 d and became detached after 7 d. For both wrapped and precipitated o-MWCNTs, the changes of elemental contents were complicated, implying the oxidization and detachment of the sp^2^ layer of CNTs. In our previous study of graphene, we observed the addition of oxygen atoms to reduced graphene oxide (RGO) by XPS [40]. No variation trend was found in RGO, because RGO should be a monolayer, whereas MWCNTs have multiple layers.

### 3.3. Raman Spectroscopy Analyses of MWCNTs after Transformation

**Figure 4:**
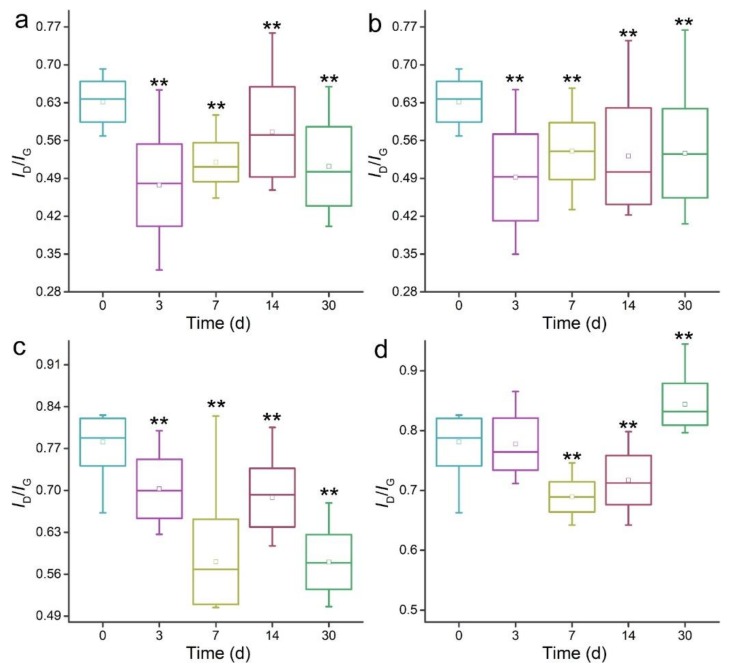
Raman spectra of MWCNTs after the incubation with *P. chrysosporium*. (**a**) Wrapped p-MWCNTs; (**b**) precipitated p-MWCNTs; (**c**) wrapped o-MWCNTs; (**d**) precipitated o-MWCNTs. Box-and-whisker plot shows the minimum and maximum (whisker bottom and top), first and third quartiles (box bottom and top), median (line inside box), and mean (square inside box) of *I*_D_/*I*_G_ (intensity of D-band/ intensity of G-band) values (*n* = 20). ** *p* < 0.01 when compared to the 0 d samples.

Raman spectroscopy could identify the defects on MWCNTs by monitoring the *I*_D_/*I*_G_ (intensity of D-band/ intensity of G-band) values. Due to the chemical oxidation, o-MWCNTs had larger *I*_D_/*I*_G_ values than p-MWCNTs (Figure 4). The *I*_D_/*I*_G_ values decreased for all MWCNTs after the degradation by *P. chrysosporium*. For wrapped p-MWCNTs, the *I*_D_/*I*_G_ values decreased at 3 d, increased at 7 d and 14 d, and decreased again at 30 d. The change of *I*_D_/*I*_G_ values should be attributed to the oxidation and detachment of carbon atoms in defects. For precipitated p-MWCNTs, the *I*_D_/*I*_G_ values decreased at 3 d and increased at 7 d. The values remained nearly unchanged at 14 d and 30 d. For wrapped o-MWCNTs, the trend was the same as for the wrapped p-MWCNTs. For precipitated o-MWCNTs, the *I*_D_/*I*_G_ values remained unchanged at 3 d and decreased at 7 d and 14 d. Surprisingly, the *I*_D_/*I*_G_ values at 30 d were larger than the initial values, suggesting that more defects were induced at 30 d.

The detachment of defects on the carbon skeleton has also been reported by other groups. The *I*_D_/*I*_G_ values increased after the degradation of SWCNTs by HRP–H_2_O_2_ [16], but the functionalized CNTs had lower *I*_D_/*I*_G_ values after the degradation in HRP–H_2_O_2_ systems at 10 d and 30 d [20]. Russier et al. attributed the decrease of *I*_D_/*I*_G_ values of o-SWCNTs after the degradation in HRP–H_2_O_2_ systems to the fact that the nanotubes with the highest amount of defects are degraded first [34]. However, increases of the *I*_D_/*I*_G_ values of o-MWCNTs were also reported in their study. Zhang et al. reported the increases of the *I*_D_/*I*_G_ values of pristine SWCNTs from 0.13 at 1 d to 0.24 at 16 d after the transformation by MnP, which were attributed to the holes and defects [36]. The oxidized SWCNTs showed great *I*_D_/*I*_G_ value increase after the degradation by lactoperoxidase for 70 h and 120 h, while pristine SWCNTs did not [35]. Bhattacharya et al. used myeloperoxidase to degrade the polyethylene glycol (PEG)-functionalized MWCNTs. Meaningful increases of *I*_D_/*I*_G_ values were observed after 7 d. Similarly, primary human neutrophils also degraded oxidized and PEGylated SWCNTs efficiently with *I*_D_/*I*_G_ value increases [41]. The increases of *I*_D_/*I*_G_ values were identified in MWCNTs transformed by *Mycobacterium vanbaalenii* PYR-1 [37]. Kagan et al. observed the increases of *I*_D_/*I*_G_ values of SWCNTs after the degradation by macrophages [38]. Bussy et al. used Raman spectroscopy to investigate the degradation of MWCNTs by microglia. Sequential increase and decrease of the *I*_D_/*I*_G_ values were assigned to the detachment of defects and the oxidation of intact surfaces [42]. Nunes et al. found the *I*_D_/*I*_G_ value decreases of MWCNTs in brain cortex [39]. Regarding the literature results, we conclude that the decreases of *I*_D_/*I*_G_ values in our study correspond to the loss of defects, and the increases thereafter should be attributed to the newly emerged intact surface.

### 3.4. IR Analyses of MWCNTs after Transformation

**Figure 5:**
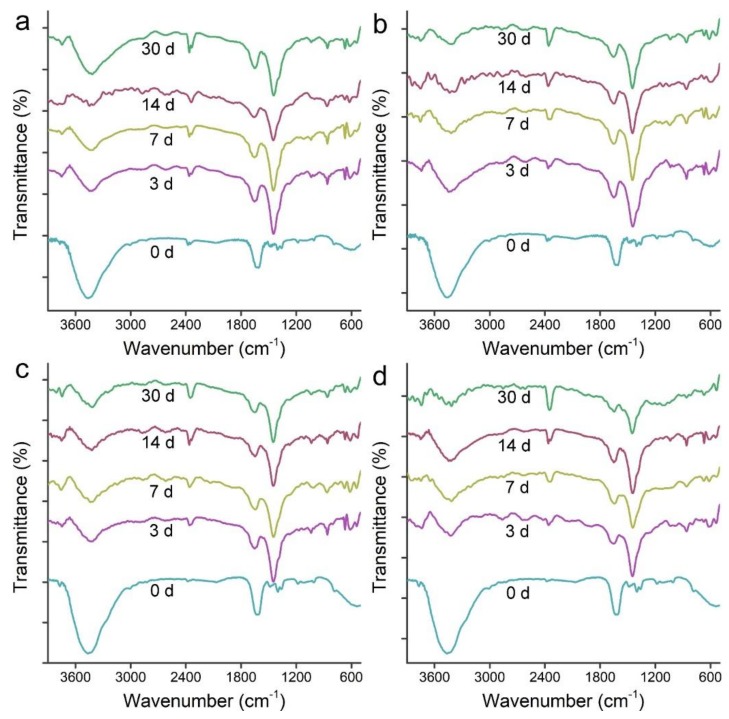
IR spectra of MWCNTs after the incubation with *P. chrysosporium*. (**a**) Wrapped p-MWCNTs; (**b**) precipitated p-MWCNTs; (**c**) wrapped o-MWCNTs; (**d**) precipitated o-MWCNTs.

The IR spectra reflect the functional groups on MWCNTs. Before transformation, p-MWCNTs showed a broad band at 3466 cm^−1^, suggesting the presence of –OH groups on the surface (Figure 5). The strong peak at 1645 cm^−1^ was assigned to aromatic C=C bonds. After transformation, even at 3 d, a tiny peak could be roughly distinguished from the 1645 cm^−^^1^ peak at 1745 cm^−^^1^ in wrapped p-MWCNTs, corresponding to the C=O bonds. Around 1450 cm^−^^1^, a strong peak emerged after the transformation, which was attributed to O–H bonds. The weak peak at 860 cm^−^^1^ was observed at 3 d and after, which was also the δ_O-H_. Similar IR peak changes were observed for precipitated p-MWCNTs. The emergence of C=O and O–H peaks indicated the addition of oxygen groups to p-MWCNTs. For o-MWCNTs, the situation was nearly the same in that the peaks for C=O and O–H became more recognizable. In the literature, the IR signals of most interest were the S2 (1000–1100 cm^−^^1^) and M1 (650–750 cm^−^^1^) bands in SWCNTs. Allen et al. observed the loss of S2 and M1 bands after the degradation of SWCNTs by HRP–H_2_O_2_ for 16 weeks [16]. Zhang et al. observed the disappearance of the S2 and M1 bands of pristine SWCNTs upon the transformation by MnP in IR spectra [36]. Similar results were reported by Kagan et al. during their investigation of SWCNTs in oxidative degradation systems [38]. Decreases of the S2 band were observed after the degradation of PEGylated SWCNTs by primary human neutrophils [41]. In our study, we used MWCNTs, and therefore we did not focus on the S2 and M1 bands. Nevertheless, the IR changes did confirm the biotransformation of MWCNTs by *P. chrysosporium*.

### 3.5. Transformation Mechanism

**Figure 6:**
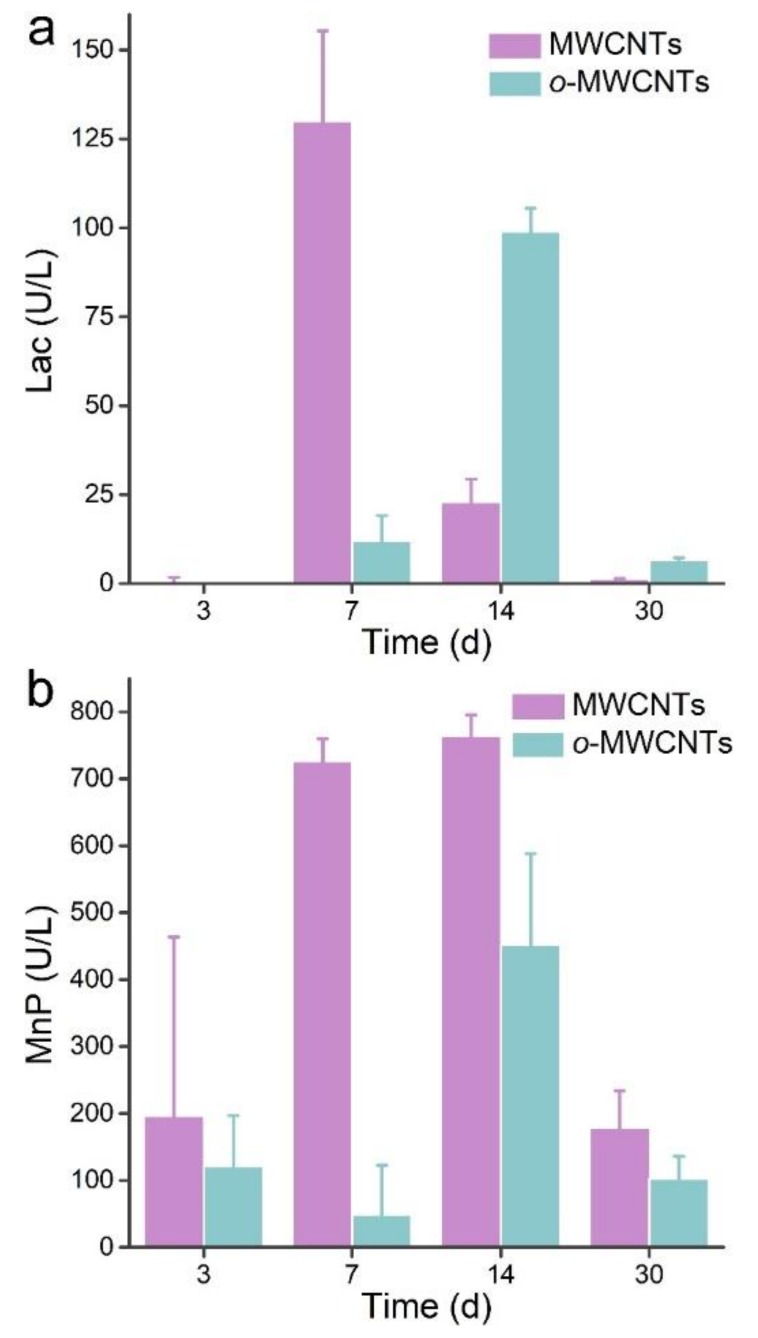
Laccase (Lac) activity (**a**) and manganese peroxidase (MnP) activity (**b**) of *P. chrysosporium*.

The production of oxidative enzymes is the most important function of white rot fungi in the oxidation and degradation of lignin and organic pollutants. Here, we quantified the enzymatic activities of Lac and MnP to reveal the transformation mechanism (Figure 6). LiP was not analyzed, because the current system only produced Lac and MnP [33,43,44]. As indicated in Figure 6a, *P. chrysosporium* exposed to p-MWCNTs produced detectable Lac activity at 7 d. The Lac activity decreased at 14 d and became very small at 30 d. For o-MWCNTs, the Lac activity became detectable at 7 d and the maximum was observed at 14 d. The Lac activity was slightly higher than in the p-MWCNT groups at 30 d, but much lower than that at 14 d. The p-MWCNT groups produced more MnP during the 30 d observation period than the o-MWCNT groups. Both groups produced meaningful levels of MnP at 3 d. High MnP activities were observed in the p-MWCNT groups at 7 d and 14 d. The max MnP activity of the o-MWCNT groups was observed at 14 d. MnP activities of both groups decreased at 30 d, just as for Lac activities. Obviously, the excretion of enzymes by white rot fungi followed a time-dependent trend, which was widely reported in the literature. The Lac and MnP were able to generate oxidative radicals to oxidize MWCNTs, which should be the main mechanism of biotransformation here. The previous studies have clearly indicated the importance of radicals for the biotransformation of carbon nanomaterials [18,31,45,46,47]. In Fenton or Fenton-like systems, H_2_O_2_ was decomposed to generate hydroxyl radicals to degrade carbon nanomaterials [19,31,45,46]. For the degradation by microorganisms and mammalian cells, the biotransformation was less efficient and slower. The H_2_O_2_ concentrations were lower in biosystems and the enzyme amounts were smaller, and therefore the incomplete degradation of carbon nanomaterials was expected [22,25,27].

The incomplete biotransformation of MWCNTs by *P. chrysosporium* has significant environmental implications. First, the long-term persistence of carbon nanomaterials in the environment would induce long-term effects. Most current environmental safety evaluations are performed within months, which could not reflect the real risks of carbon nanomaterials in the environment. Second, despite the stability of MWCNTs against biotransformation, partial oxidation and shortening occurs. The addition of oxygen-containing groups to the carbon nanomaterial surface would change their environmental transportation and also the bioeffects [33,43,48]. Thus, the transformation of carbon nanomaterials should be considered when designing experiments to evaluate their environmental behaviors and effects. Third, the stability of MWCNTs against biodegradation suggested that direct biodegradation of carbon nanomaterials was not efficient enough for remediating them. Harsher conditions should be adopted to decompose them before discharging into the environment; for example, advanced oxidation (with H_2_O_2_ and/or UV) of functionalized carbon nanotubes (CNT-OH and CNT-COOH) and its influence on the stabilization of CNTs in water and tannic acid solution, and the degradation of oxidized multiwalled carbon nanotubes in water via the photo-Fenton method and its degradation mechanism [15]. However, the good stability of carbon nanomaterials may be desirable for applications in complicated systems, such as antibacterial applications, remediation applications, and agricultural applications. In the future, the molecular design to facilitate or block the biotransformation of carbon nanomaterials will be in high demand.

## 4. Conclusions

In summary, the biotransformation studies showed the oxidation and shortening of MWCNTs in *P. chrysosporium* culture systems, while the complete degradation of the tubular structure was not observed. The fungal biotransformation of MWCNTs was attributed to the enzymatic oxidation by Lac and MnP. The addition of oxygen atoms to the carbon skeleton and the detachment of defects were the main evidences of the biotransformation. The o-MWCNTs wrapped in the fungal balls showed more short tubes, suggesting the higher degree of transformation. We believe that the fungal transformation of carbon materials has significant environmental importance in evaluating their environmental risks and safety. The relative stability of carbon nanomaterials against biodegradation by microorganisms reminds us to pay more attention to the long-term environmental effects of these carbon nanomaterials.
